# Visualizing dynamic data with heat triangles

**DOI:** 10.1007/s12650-021-00782-y

**Published:** 2021-09-01

**Authors:** Ya Ting Hu, Michael Burch, Huub van de Wetering

**Affiliations:** Technische Universiteit Eindhoven, Eindhoven, The Netherlands

**Keywords:** Dynamic data, Statistics, Data aggregation, Interaction techniques, Visual design, Information visualization

## Abstract

In this paper, an overview-based interactive visualization for temporally long dynamic data sequences is described. To reach this goal, each data object at a certain time point can be mapped to a number value based on a given property. Among others, a property is application-dependent and can be number of vertices, number of edges, average degree, density, number of self-loops, degree (maximum and total), or edge weight (minimum, maximum, and total) for dynamic graph data, but it can as well be the number of ball contacts in a football match, or the time-dependent visual attention paid to a stimulus in an eye tracking study. To achieve an overview over time, an aggregation strategy based on either the mean, minimum, or maximum of two values is applied. This temporal value aggregation generates a triangular shape with an overview of the entire data sequence as the peak. The color coding can be adjusted, forming visual patterns that can be rapidly explored for certain data features over time, supporting comparison tasks between the properties. The usefulness of the approach is illustrated by means of applying it to dynamic graphs generated from US domestic flight data as well as to dynamic Covid-19 infections on country levels.

**Graphic abstract:**

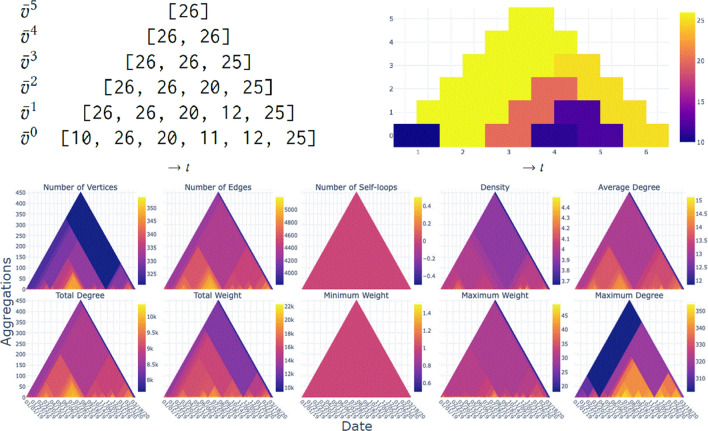

## Introduction

Exploring dynamically changing data is a challenging task due to the fact that the data can consist of many time steps, and each data object at a certain time point can be too complex to be visualized as several time-varying variations. For example, a dynamic graph (Beck et al. 2017; Vehlow et al. 2015), consists of a sequence of static graphs, for which the generation of a visual overview becomes a challenging research question, even more if the sequence contains several hundreds or thousands of graphs (Burch et al. 2017a). Getting an overview about temporal patterns in such a temporally long data sequence is difficult by traditional visualization techniques, typically trying to show the data structures in its entirety. A more condensed visualization combined with interactions is required as a first stage to provide an overview, allowing to start further exploration processes and to guide an observer to build hypotheses about the data dynamics. A second stage, serving as details-on-demand, provides more insights into the data structures, typically showing a shorter time period.

On the one hand, visualization techniques can be a more effective and appropriate way to represent the data objects and their changes over time than given numbers that summarize those properties. However, on the other hand, the bigger the amount of data, the more useless a fully-fledged dynamic data visualization may become due to visual clutter (Rosenholtz et al. 2005), making it difficult to spot the differences over time.

There is no suitable alternative that shows temporally long data sequences while providing at the same time an overview of the dynamic properties in any of the subsequences. In particular for the field of dynamic graph visualization, there is a list of approaches (Beck et al. 2017) that do not provide a more aggregated perspective on those subsequences.

To mitigate this challenge while finding patterns in the dynamic data, we provide a scalable overview conveying properties of the data to start further exploration processes. Interactions then allow to navigate and to decide where the focus should lie on. This overview-and-detail approach might lead to finer details and structures, possibly finding what one is interested in.

**Fig. 1:**
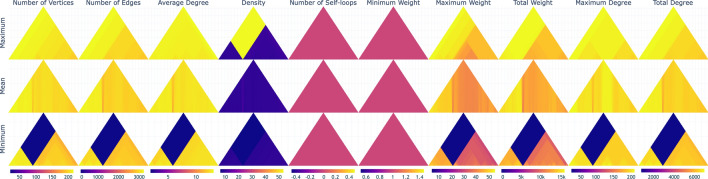
US domestic flights from 2001 to 2002: Heat triangles based on property (columns) and aggregation strategy (rows). Columns have a color legend for their actual value range; for single value ranges that value is in the middle of the legend

The main goal of this work is to give an overview of long data sequences that can be used as a means to interact with, to navigate, or to identify temporal subsequences of interest. We introduce the **heat triangle**, a novel visual representation and navigation model for dynamic data (see Fig. 1).

To reach this goal, we focus on reducing each data object at a certain time point from the sequence to a certain well-defined real number based on a property $\pi$. We further aggregate these numbers by aggregation operators. Interactions can be applied to modify the heat triangle views. We illustrate the usefulness by means of application examples based on a US domestic flight traffic dataset (United 2020) and a dynamic Covid-19 dataset containing time-varying infection numbers on a country level.

This work is an extension of a formerly published paper at the international symposium on visual information communication and interaction (VINCI) (Hu et al. 2020). We provide several more add-ons compared to the original work which are listed in the following.

- *General dynamic data* Apart from restricting ourselves to graph data, we extend the work to general dynamic data which consists of time-varying data objects.
- *Extended data model* We extended our data model to fit to general dynamic data which is transformed into time-dependent numbers that represent each data object over time (Sect. 3.1).
- *Interaction techniques* We provide many more details on interaction techniques by focusing on the work by Yi et al. (2007) taking into account typical categories that are relevant for our approach (Sect. 5).
- *Application example* Apart from just focusing on dynamic graph data, we also take a look into an application example investigating time-varying Covid-19 infection numbers (Sect. 6.3.)
- *Discussion and limitations* We give a discussion on limitations and further possibilities to incorporate aggregation metrics to derive even more insights from the dynamic data (Sect. 7).

To sum up, a visual way is introduced to get an overview about long time-varying data sequences by first projecting each data object to a certain well-defined number. As a second step, a temporal aggregation is introduced while the resulting values are visually encoded in a certain color. The generated visual patterns look like color coded triangles, with the apex of each subtriangle indicating the aggregated value of the underlying temporal subsequence.

## Related work

Visualizing dynamic data can be done in two major ways, either as a static representation or an animation (Aigner et al. 2011). An animated diagram can show all the data objects in its entirety but only one at a time leading to comparison problems (Tversky et al. 2002), but the data visualization is not limited from the perspective of the display capacity. If a static approach is used instead, only a few instances in time of the dynamic data can be visually represented, but it would be beneficial to get an overview about the complete dynamics as an overview to start further explorations (Burch et al. 2017b). Aggregation is a powerful technique, but it mostly focuses on showing the structure of the underlying data object that is changing over time. In our approach, we provide a method to transform each data object to a real value and aggregate those values over time to show the differences and commonalities in the dynamic periods. If interesting time spans are identified we support interaction techniques (Yi et al. 2007) to dig deeper in the dynamic data, also showing more structures on the individual data objects.

In particular, for the field of graph visualization, there are two major ways to visualize static graphs (Landesberger et al. 2011): node-link diagrams and adjacency matrices (Burch 2010). However, for a dynamic graph (Beck et al. 2017) visualization is always problematic since a lot of display space is required to show the dynamics of the graph data, no matter which kind of metaphor is chosen which is a general problem for dynamic data, not only dynamic graph data. From a perspective of the static data, i.e., for data objects at a certain time step, node-link diagrams suffer from visual clutter (Rosenholtz et al. 2005) while adjacency matrices are not suitable for path tracing tasks (Ghoniem et al. 2005), hence it would be a valid solution to first show the entire dynamics of the data sequence on a value-aggregated view that guides the observer to certain interesting time periods first, also allowing to pick several of them to compare the different time periods side-by-side.

Moreover, also the attributes of dynamic graphs can vary over time, which adds a level of complexity to the data representation problem. Time becomes an important data dimension that can reveal trends in dynamic patterns and relationships, for example to explore varying community structures (Dang and Nguyen 2018; Wang et al. 2018). Visualizing dynamic data and also dynamic graphs, as an example for such complex dynamic data, are challenging due to at least three major data dimensions of interest: vertices, edges, and time (Bruder et al. 2018; Burch 2010), while general dynamic data can consist of any kind of extra attributes worth encoding visually.

Animation techniques or static charts based on a timeline have been implemented for dynamic graphs (Beck et al. 2017). The former has some drawbacks as the user not only has to concentrate and pay good attention during the animation but also needs to memorize the graph shown per time step which may put a significant load on the cognitive efforts that the user has to make (Burch 2010). The understanding has to remain consistent throughout the animation to prevent any confusion (Purchase et al. 2006). Especially for long graph sequences, this becomes problematic because the complete sequence of graphs can typically not be shown for vertices, edges, and all time steps.

According to Kerracher et al. (Kerracher et al. 2015), the design space can be split into two dimensions namely (1) task categories (lookup, comparison, and relation seeking), and (2) the data items involved (elements and structures of the graph in single or multiple time steps). With our approach, we cannot support all these tasks but we can provide a scalable overview of the time aspect, providing a good starting point for hypotheses building and further data explorations. Hence, we follow the visual information seeking mantra (Shneiderman 1996), starting with graph abstractions in the form of representative values.

With the use of interactions, such abstractions can act as a powerful mechanism by selecting properties the user wants to focus on, leaving out the ones which are irrelevant at the moment (Rafanelli et al. 1995). The heat triangle then acts as an overview showing the change in time for each selected property. Node-link diagrams or a suitable visual metaphor for general data objects can act as a details-on-demand feature providing an in-depth view on the elements and structure of the graphs or general data objects of the selected time steps.

## Dynamic data

Static data are mostly easier to visualize since it is just based on one instance in time, i.e., the entire display space is available to visually encode the data object. For dynamic data, on the other hand, we have several time-varying instances of a data object, hence we have to find an alternative for the visualization of as many time steps as possible, mostly at first place fulfilling the purpose of providing an overview over time, while the structure of the data is degraded to a second, but not minor role. Such an overview on all possible time periods in the entire sequence can be obtained by first transforming each data object to a well-defined number based on a certain data-contained and user-defined property.

### Data properties and aggregation

A general (static) data object might be mathematically modeled as an object *O* with attached data attributes denoted as a finite list by $a_1,\ldots ,a_n$. Such a data object *O* can have a time-varying nature resulting in an object sequence

$$\begin{aligned} {\tilde{O}}:=(O_1,\ldots ,O_m) \end{aligned}$$

A certain data property $\pi$ might be of particular interest which has to be observed over time and for which an overview is required in all of the subsequences, i.e., the entire time dimension is split into time periods of any length to allow comparisons of the time periods, also over longer temporal distances. To reach this goal, we first support the computation of the property $\pi$ for each of the individual data objects. For this to do successfully, we need some kind of well-defined formula with which we can derive a value for each object individually. The final step is to compute aggregation values for each of the subsequences based on the property values and some standard aggregation operator $\sigma$ like mean, minimum, or maximum. The next section describes the general idea of a data object by focusing on dynamic graph data which has some complexity due to the fact that each graph (data) object consists of vertices, edges, and extra data attributes.

### Excursus and example: graphs

We denote a graph *G* as a pair $G=(V,E)$ for which the non-empty vertex set is $V=V(G)=\{w_1,\ldots ,w_n\}$ , and the edge set $E=E(G)$ is a collection of unordered pairs of vertices $(w_i,w_j)$ if the graph is undirected and ordered if the graph is directed. For the sake of simplicity, we will only focus on directed graphs, but in general there is no limitation to also consider undirected graphs.

The degree of a vertex *v* is the number of edges incident to it, that is,

$$\begin{aligned} d(v)=|\{w|(v,w) \in E\}| + |\{w|(w,v) \in E\}|. \end{aligned}$$

The total number of self-loop edges (*v*, *v*) in a graph equals

$$\begin{aligned} \sum _{v\in V} s(v), \,\,\text { where }\,\, s(v)=|E \cap \{(v,v)\}|. \end{aligned}$$

The density of a graph measures how many edges are in set *E* compared to the maximum possible number of edges between vertices in set *V*. The density of *directed* graphs is defined as

$$\begin{aligned} D=D(G)=\frac{|E|}{|V|^2}. \end{aligned}$$

Lastly, the average degree of a graph is calculated by

$$\begin{aligned} K=K(G)=\frac{|E|}{|V|}. \end{aligned}$$

A dynamic graph ${\widetilde{G}} = (G_1, \ldots , G_m)$ is a sequence of *m* graphs, where $G_t=(V_t,E_t)$ represents the instance of any time step *t*.

**Fig. 2:**
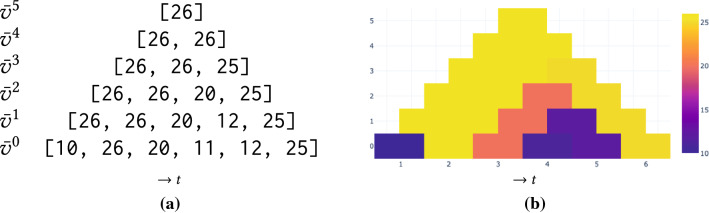
Aggregation and visualization: (**a**) property tuples: the original tuple at the bottom and aggregations with the maximum operator on top of it. (**b**) corresponding heat triangle: horizontally the time axis and vertically the aggregation level

Given dynamic graph ${\widetilde{G}}$, our approach involves a graph property $\pi$ that maps the static graphs $G_i$ to a number, e.g., vertex count and an aggregation operator $\sigma$ that aggregates a sequence of numbers into a single number, e.g., the maximum operator. Consider tuple ${{\bar{v}}} = [v_1,...,v_m]$ with $v_i=\pi (G_i)$, the value of property $\pi$ for graph $G_i$. We are interested in the aggregated values obtained by operator $\sigma$ for each possible subsequence $[v_i, ..., v_{i+k}]$ of ${{\bar{v}}}$ and organize them, for $0\le k<m$, in aggregation tuples

$$\begin{aligned} {{\bar{v}}} ^k = [ \sigma (v_1, ..., v_{1+k}), \sigma (v_{2}, ..., v_{2+k}), ..., \sigma (v_{m-k}, ..., v_m)]. \end{aligned}$$

For example, for the tuple ${{\bar{v}}}=[10,$ 26,  20,  11,  12,  25] with $m=6$ values and using the maximum operator for aggregation, Fig. 2a shows the aggregation tuples ${{\bar{v}}}^0$, ..., ${{\bar{v}}}^5$ in which, for example, the value 25 in ${{\bar{v}}}^3$ corresponds to the maximum in the subsequence [20, 11, 12, 25] of the original tuple ${{\bar{v}}}={{\bar{v}}}^0$.

Figure 2b illustrates a visualization of the aggregation tuples by means of a heat triangle: we replace the numbers in the tuples by $1\times 2$ rectangles that are horizontally aligned in stacked and centered rows. The rectangles are filled using an appropriate color map. Visualized in this way, each rectangle is the apex of a triangle and represents the aggregated value for the subsequence of the original tuple at the base of this triangle. All cells together represent the aggregated values for all possible subsequences.

The current implementation has three aggregation operators, namely mean, minimum, and maximum; furthermore, the following properties are supported: number of vertices |*V*|, number of edges |*E*|, number of self-loops $\sum _{v\in V} s(v)$, density in percentages $D\cdot 100\%$, average degree *K*, total degree $\sum _{v\in V} d(v)$, maximum degree $max\{d(v)|v\in V\}$, and for graphs with edge weights, total amount of weight, minimum amount of weight, and maximum amount of weight.

## Heat triangles

In this section, we describe several ways that the generated heat triangles can be used.

**Fig. 3:**
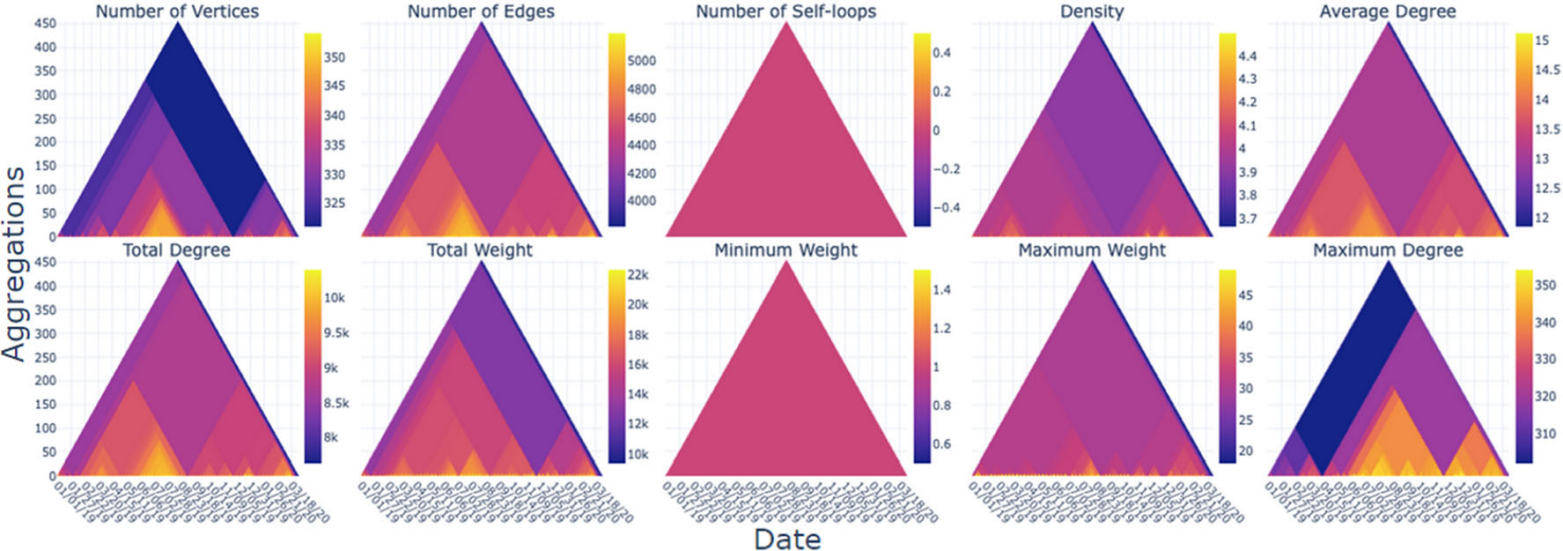
Multiple heat triangles view of the US domestic flights from January 2019 to March 2020 of each property aggregated by minimum value

### Multiple heat triangles

Heat triangles can be shown per property (see Fig. 3). The time steps are given on the x-axis, the level of aggregation is given on the y-axis, and the colors represent the values of the properties at a specific time step, and level of aggregation with a corresponding color bar representing the range of the values.

### Two heat triangles

**Fig. 4:**
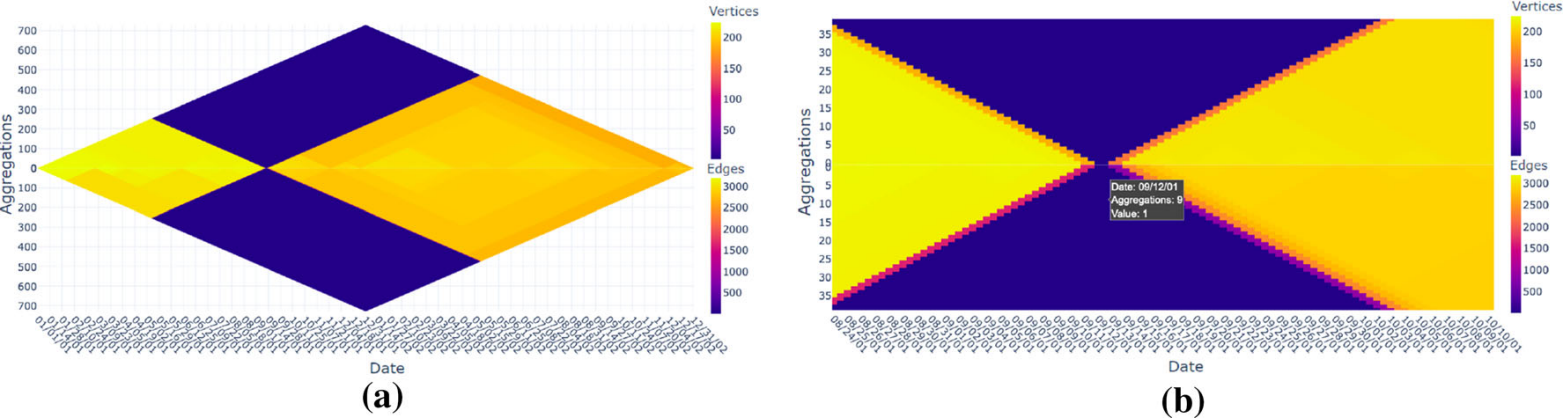
Two heat triangles view for the number of vertices and edges aggregated based on minimum showing that there is a drop around September 2001 in both (**a**). Hovering on the above heat triangle gives the value of the number of vertices, e.g., zooming-in on September 12, 2001, there are only 2 vertices and 1 edge (**b**)

For comparison of either two properties with the same aggregation method or two aggregation methods for the same property, two juxtaposed (Gleicher et al. 2011) heat triangles, one of which is vertically flipped, can be used (see Fig. 4). The heat triangles share the same x-axis to easily compare the two in detail over time.

**Fig. 5:**
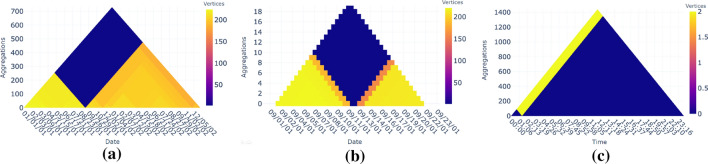
One heat triangle view: (**b**) A sub-heat triangle is created from (**a**) with the clicked point in the heat triangle aggregated based on minimum of vertices as the new peak. The number of vertices is around 200 at the beginning of September 2001, decreases on September 11, and decreases further until 2 vertices on September 12. It starts to increase on September 13 and increasing more on September 14. (**c**) A nested heat triangle with minutes as the new time step is created with the clicked point in the heat triangle aggregated based on maximum of vertices based on the time step. The one flight found during September 12 takes place around 1:30 a.m. whereas all other flights are canceled. The flight is between the airports Los Angeles, CA and Houston, TX

### One heat triangle

Compared to the previous views, which mostly give an overview or allow the user to view relationships between the heat triangles, the view shown in Fig. 5 allows to go into detail for one chosen property and method of aggregation.

#### Sub heat triangle

The sub-heat triangle in Fig. 5b is a heat triangle with as its pinnacle the selected cell in the main heat triangle shown in Fig. 5a. This way the user can zoom-in into a heat triangle to focus on relevant time periods.

#### Nested heat triangle

Figure 5c shows a nested heat triangle, which is created based on the time step of a clicked cell in the main heat triangle. The time step used for the main heat triangle is namely grouped per day, whereas the time step for the nested heat triangle is grouped either by the minute or by the hour for the corresponding day. This allows the user to see the change throughout a specific day instead of throughout a period of multiple days.

### Detail view

The user has the opportunity to compare two node-link diagrams per different time steps like some kind of detail view. To this end, we support several node-link diagram layouts like circle, distributed recursive (Martin et al. 2007), Fruchterman-Reingold (Fruchterman and Reingold 1991), Kamada-Kawai (Kamada and Kawai 1989), large graph, random, and Reingold-Tilford tree (with and without polar coordinate post-transformation) (Reingold and Tilford 1981). We do not show these node-link diagrams in this paper since they are not a novel contribution. However, they are important as a details-on-demand feature and starting from there, many more interaction techniques could be incorporated.

### Two nested heat triangles

**Fig. 6:**
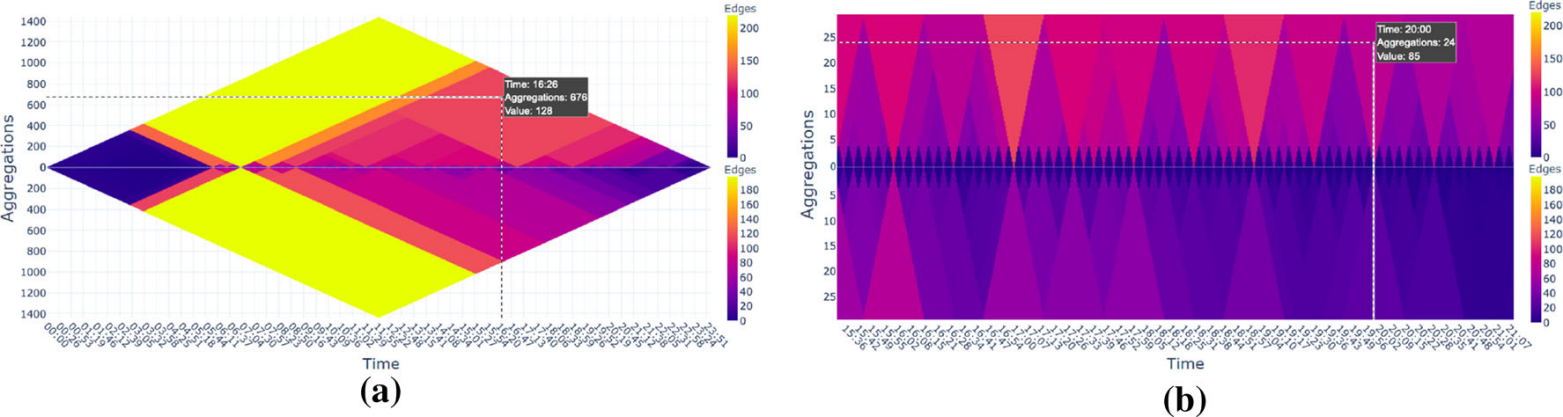
(**a**) Two nested heat triangles view: Two heat triangles of the number of edges aggregated based on minimum with minutely time step on September 9, 2001 (above) and November 28, 2002 (below) showing similar patterns throughout the day, but the numbers in 2002 are lower. (**b**) The pattern throughout the day is almost the same, even zoomed-in. This is due to airports following certain time schedules for all flight departures

Lastly, instead of having one nested heat triangle, which depends on the click value based on users’ demands, also two nested heat triangles are possible, which depend on two user inputs as shown in Fig. 6. This provides the user to give two dates as input to produce two nested heat triangles in the same layout as the two heat triangles in one of the previous views to compare the change throughout the day with one another. Also here, the user can choose graph properties, methods of aggregation, time step, color scheme, and range of time.

**Fig. 7:**
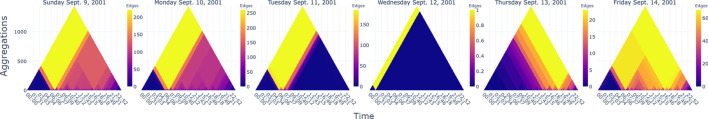
Chronological view: Heat triangles for the number of edges aggregated by maximum with time step minutes from September 9, 2001, to September 14, 2001. The heat triangles of the first two days are similar to each other, whereas on Tuesday there has been a decrease throughout the day until a complete stop of flights. The next day there is only one flight spotted around 1:30 a.m. whereas all other flights are canceled. On Thursday, some flight connections have been resumed. The number of flight connections on both Thursday and Friday is still drastically lower compared to the days before September 11, 2001

## Interaction techniques

We follow the taxonomy presented by Yi et al. (Yi et al. 2007) to implement a list of suitable interaction techniques.

- *Select* We allow the selection of visual elements to get a details-on-demand in form of a tool tip for example, or to annotate and highlight interesting elements.
- *Explore* Time periods can be modified on users’ demand, for example to move from one period to another one, leading to an exploration of the data over time. This is in particular useful, if not all time steps are shown due to a zoomed-in temporal level.
- *Encode* We support several options to show the output of the aggregation operations visually. One of those is by modifying the color scales which could be done by each user individually. Another option to encode the data is by the different heat triangle arrangements for example.
- *Abstract/Elaborate* We allow zooming on different levels of temporal granularity. Moreover, as some kind of semantic zooming we support the details-on-demand feature that shows the graph data in its original form, for example as a node-link diagram in a certain layout.
- *Reconfigure* The order of the heat triangles can be modified to place those next to each other that might have similarities in the visual patterns. The perceptual abilities of the human’s visual system help to do this reliably, however, a more automatic solution is desirable.
- *Filter* Since the aggregation operators compute real numbers we can even filter on those numbers to see which visual patterns remain, reflecting a certain dynamic data pattern. We might even filter for data-related aspects before the aggregation is computed.
- *Connect* If several heat triangles are shown or another view, for example, a node-link diagram for the graphs, we can select certain visual elements, and the selected ones are then highlighted in all of the views.

## Application examples

To test the overall usefulness and usability of heat triangles on dynamic graph data while demonstrating the scalability of the technique for the number of edges, US domestic flight data are separately extracted from the years 2001-2002 and 2019-2020, and the canceled flights are discarded. These two particular time frames are chosen due to the 9/11 attacks and the Covid-19 pandemic which took place in the years 2001-2002 and 2019-2020, respectively, impacting US domestic flight traffic drastically. In Sect. 6.3, the heat triangles are applied to non-graph dynamic data, namely the number of detected Covid-19 contaminations per Dutch city per day in a period of approximately a year starting from February 28, 2020.

### US domestic flights from 2001 to 2002

The flight data from the years 2001 and 2002 consist of 10,942,798 rows, 730 time steps (=days), 153,915 vertices, and 2,170,496 weighted edges. In Fig. 1, the heat triangles are shown for each property and aggregation strategy.

For this dataset vertices correspond to airports, edges to flight connections, and the edge weight is the number of flights for a flight connection. The maximum/minimum weight is therefore the maximum/minimum number of flights, and the total weight is the total number of flights on a flight connection.

The density describes the portion of the potential flight connections in a network that are actual connections. The average degree is the average number of flight connections per airport. The degree depicts the amount of incoming and outgoing flights per airport, and therefore, the total degree is the total amount of incoming and outgoing flights, and the maximum degree is the maximum of incoming and outgoing flights for some airport. All properties are calculated per time step, that is, per day.

#### Overview

We can see that in Fig. 1 the heat triangles for the number of self-loops and minimum weight are both one-colored. Hovering over the heat triangle indicates a value of 0 for the number of self-loops and a value of 1 for minimum weight. Having no self-loops indicates that there is no flight with the same airport as both departure and arrival destination, which is as expected.

The minimum weight of value 1 indicates that the number of flights between airports is at least one. The value cannot be equal to zero, because airports without any flight connections are not included in the network. This means, however, that for every day in the period 2001-2002 there exists at least one flight connection that has precisely one flight on that day.

For the other heat triangles, there is a noticeable color variation. Already from the last row in Fig. 1, where each heat triangle is aggregated by minimum, it can be concluded that there has been a drop in the number of airports, the number of flight connections, the average degree, the maximum and total number of flights, the maximum and total degree, and slightly in the density. Striking is that the density, when aggregated by maximum, is the largest value around the same period. For this to occur either the number of edges drastically increased or the number of vertices drastically decreased.

As a result, the multiple heat triangles view draws the attention of the user to a specific period. Depending on the context, it will be decided on which property or properties the focus will be laid on.

**Fig. 8:**
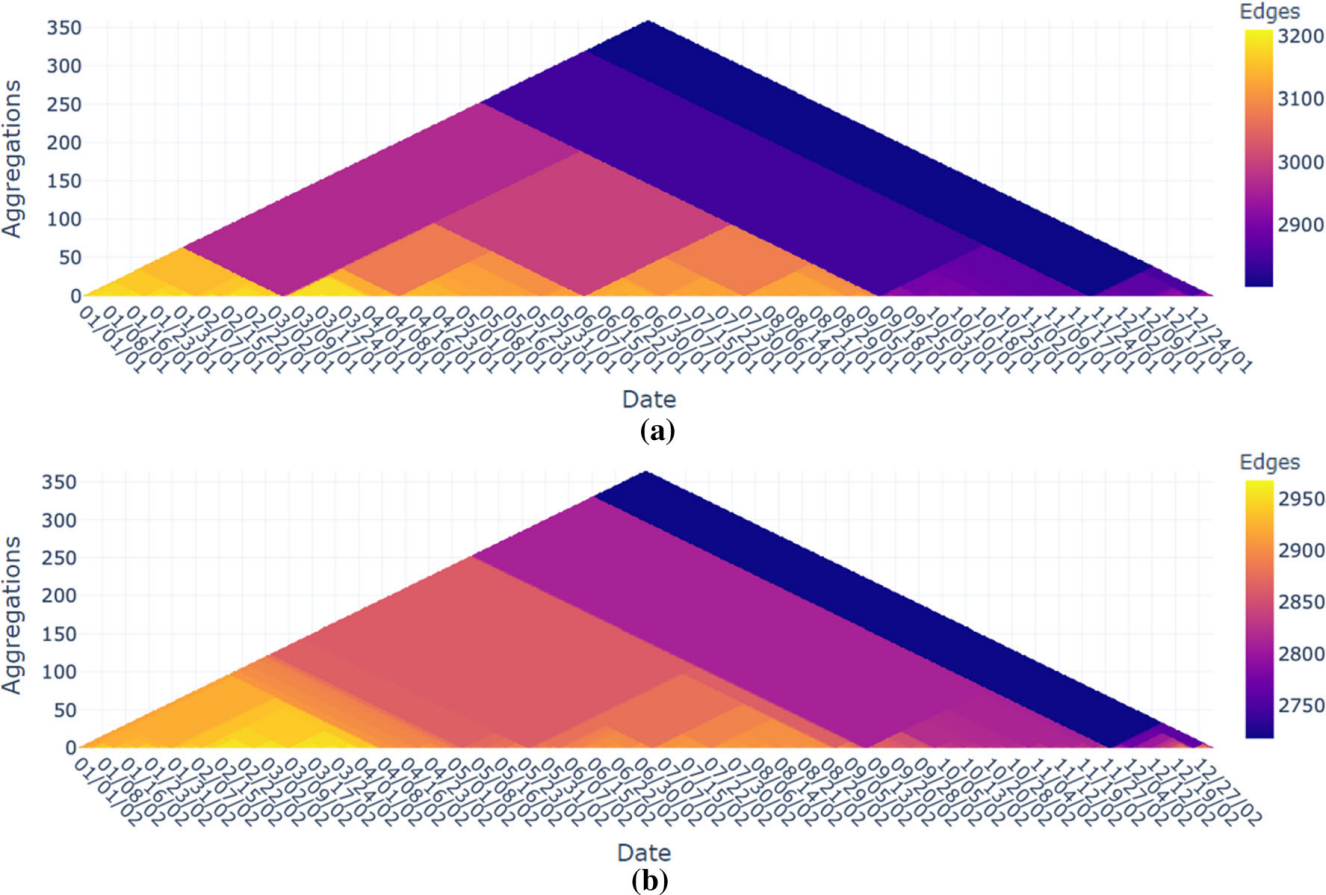
Heat triangle for the number of edges aggregated by minimum for (**a**) the year 2001 after filtering out the days around the event of 9/11 and (**b**) the year 2002. It shows that the least number of flight connections excluding the days around 9/11 occur for the year 2001 during Thanksgiving (2802), the day after Thanksgiving (2819), and Christmas day (2,851). For the year 2002, the least number of flight connections is also during Thanksgiving (2,718), the day after Thanksgiving (2754), and Christmas day (2,761). For all the three days, the number of edges is slightly less in 2002 compared to 2001

**Fig. 9:**
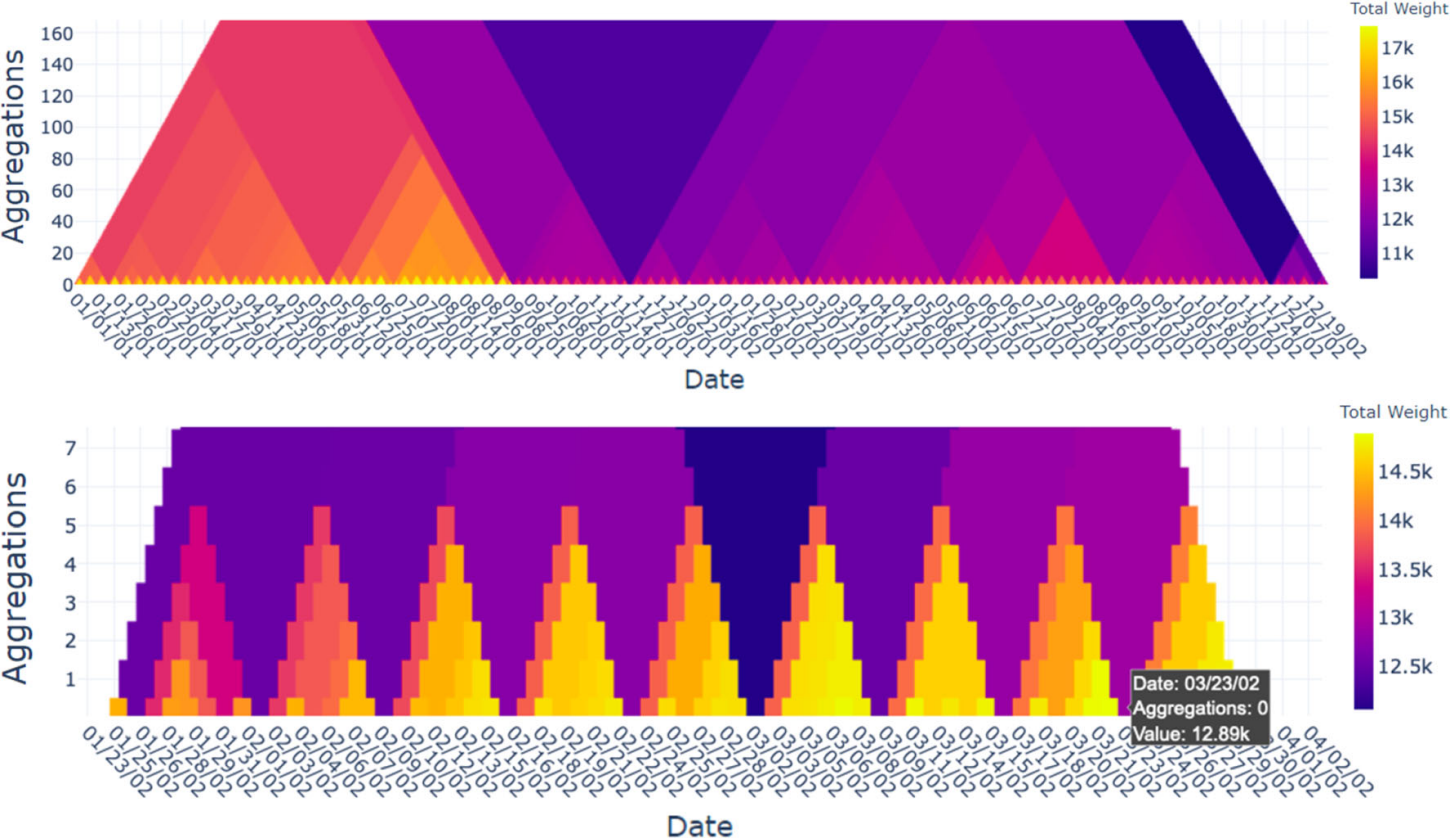
Zoomed in heat triangle for total weight aggregated by minimum (top) and a sub-heat triangle (bottom), showing a pattern throughout the entire period for higher flight counts during weekdays compared to weekends

#### Impact of 9/11

When seeing this period up close for the properties number of vertices and edges and with the help of user interactions in Fig. 5, it shows that the number of vertices drops from 220 to 186 on September 11, 2001, and to 2 vertices on September 12. The number of vertices then increases to 158 on September 13 and to 213 in the coming days. The same pattern is noticed for the number of edges, namely from 3,108 to 1,634 on September 11 and to only 1 edge on September 12. The number of edges then increases to 851 on September 13 and to 2,393 on September 14, 2,721 on September 15, 2,844 on September 16, and to around 2,900 in the coming days.

By clicking on the cell of the heat triangle, new heat triangles are created based on the time of the chosen day as time step. In Fig. 7, six heat triangles are shown for the number of edges aggregated on maximum based on the days September 9 until September 14. The first two days are similar to each other. Most flight connections take place in the morning around 7 a.m., and it slowly decreases throughout the day. The maximum flight connections per minute are higher on Monday than on Sunday. It is also possible that the flight connections become more spread out throughout the day. To see this one can use time steps of hours instead of minutes.

On Tuesday, September 11, the morning is similar to the previous two days. However, the amount of flight connections is starting to decrease drastically throughout the day and even reaches zero flight connections after 9:20 a.m. The next day, there are zero flight connections. Only around 1:30 a.m. there seems to be one flight connection that has not been canceled. This is in line with the findings from Fig. 5.

The first resumed flight is spotted on Thursday, September 13 at 5:55 a.m. slowly increasing to a maximum of 23 flights departing at 7 p.m. Also on Friday, there have been some flights with a similar pattern as the day before. However, the amount of flights for both days, especially in the morning, is still drastically less compared to before September 11, which we also saw in Fig. 5.

An explanation for this observation is the rapid grounding of air traffic across the US in response to the 9/11 terrorist attacks. On the morning of September 11, 2001, four planes were hijacked. At 9:06 a.m., the Federal Aviation Administration bans takeoffs of all flights bound to or through the airspace of New York Center, later followed by a complete ground stop to all traffic closing all US airspace for the first time in history.

If not the exact number of nodes and edges is of interest but only the relationship between the two, then one can also look at either the change in the density or the average degree. From the heat triangle of the density aggregated by maximum in Fig. 1, there is a great change in value occurring on September 11 which can be explained by the fact that there are only one edge and two vertices during this time step.

#### Patterns before and after 9/11

After this tragic incident, many changes were implemented in airport security procedures. Airports, which could not meet the new safety requirements, were shut down, and fewer flight connections were allowed. The on-going security concerns and the increased hassle factor both led to a reduction in demand for air travel and a slow rate of passenger return, eventually resulting in bankruptcy for some airlines (Blalock et al. 2007; Gittell et al. 2006).

**Fig. 10:**
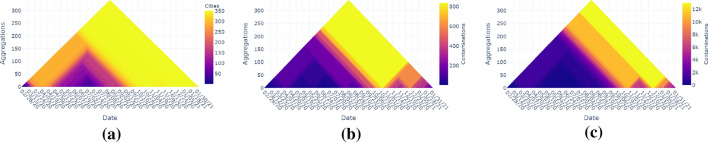
The maximum aggregation operator is useful to visually explore the number of cities with Covid-19 infections: (**a**) At least one contamination. (**b**) Number of daily contaminations in any city. (**c**) Total number of contaminations in all cities

The latter can be seen in the heat triangles in Fig. 1 as there is a slight difference in coloration indicating lower values for the number of vertices, edges, maximum and total weight, and maximum and total degree in 2002 compared to 2001. This can be made more visible by filtering out the days around the 9/11 attacks as is done in Fig. 8a by choosing the period January 1, 2001, until December 31, 2001, and discarding the lowest value in the data, i.e., effectively filtering out 9/11. The same procedure is repeated for the year 2002, but without discarding the lowest values resulting in the heat triangle shown in Fig. 8b.

The heat triangle in Fig. 8a for the number of edges aggregated by minimum then shows that November 22, 2001 (Thanksgiving) is the day with the lowest number of edges namely 2802 followed by November 23, 2001 (day after Thanksgiving) with 2819 edges, and December 25, 2001 (Christmas day) with 2851 edges. The heat triangle shows that the minimum amount of flight connections (2718) in the entire period, except for the days around 9/11, takes place on November 28, 2002.

A heat triangle for November 28, 2002, is created based on minutely time steps and shown in Fig. 6. It contains a similar pattern as the heat triangles for September 9 and September 10 in 2001, as shown in Figs. 7 and 6. However, the values for November 28 are lower. This makes sense since most people prefer to be at their destination on Thanksgiving/Christmas morning instead of at the airport.

The other patterns in Fig. 6 can be explained by the holiday season of the US. The summer holidays usually start in early June and end in early September, which can be seen in both heat triangles although for 2002 this seems to be less noticeable compared to the year before. During February, the winter recess takes place, and during April, there is a spring recess, leading to a higher number of flight connections. To check for the busiest days, it is advisable to produce the same heat triangle but aggregated by maximum instead of minimum to make this pattern more noticeable.

All these patterns are not only noticeable for the number of edges, but also for the other properties. Moreover, for the property total weight and aggregation method minimum small tiny triangles for the entire period are noticed. In Fig. 9, the view is zoomed in to clearly see this pattern. On Saturday, February 16, 2002, the total weight is equal to 12.7k. The next day this amount is equal to 13.9k, and it increases to 14.7k on Monday, February 18, 2002. The amount stays consistent for the upcoming days fluctuating around 14.6k and drops eventually to 12.7k on Saturday, February 23, 2002. Then it follows the same pattern by increasing the next day to 13.9k to being reasonably stable throughout the next five days fluctuating around 14.5k. Thus, it seems that during the weekdays the total number of flights is higher compared to the weekends. Remarkably, the number is higher on Sundays than on Saturdays. This pattern stays stable throughout the entire period except for some events and holidays.

### US domestic flights from 2019 to 2020

The flight data from January, 2019 until March, 2020 consists of 8,994,705 rows, 456 time steps (=days), 155,732 vertices, and 2,205,661 edges.

#### Overview

An overview per property aggregated by minimum is shown in Fig. 3. Similar to the US domestic flight data from 2001 to 2002, the heat triangles of the number of self-loops and the minimum weight are one-colored.

Moreover, the weekly pattern is also noticed here for the heat triangles showing the total weight and the maximum weight, meaning that the pattern which was seen in 2001 persists in 2020. The same holds for the Thanksgiving tradition, which can be spotted in the heat triangle for the number of vertices. On the other hand, the heat triangle for the number of edges shows fewer flight connections around January in 2019 than in 2001.

Even the previously mentioned holiday patterns are present such as the summer holiday (early June until early September), winter (February), and spring recess (April). From the color scales, we observe that the number of airports, the number of flight connections, and the number of flights have drastically increased over 18 years. Furthermore, derived from how much the maximum degree and average degree have increased, the world seems to have become more connected. This is beneficial for travellers, but has the negative impact that diseases and viruses like Covid-19 can be spread much faster all around the globe. To investigate the influence of Covid-19 on the flight traffic, we explore the dynamic network data for typical phenomena by applying the heat triangle visualization.

#### Impact of Covid-19 pandemic

There is, however, a significant drop noticed for the degree (average and total), density, weight (maximum and total), number of edges, and number of vertices around the end of March 2020. This drop has started on March, 25 and keeps decreasing in the next days until March, 31 which is the last time step available in the data. No special patterns throughout the day(s) are found. The heat triangle is very similar to the nested heat triangle of November 28, 2002 (see Fig. 6), but with a higher overall decrease in values in the evening hours.

**Fig. 11:**
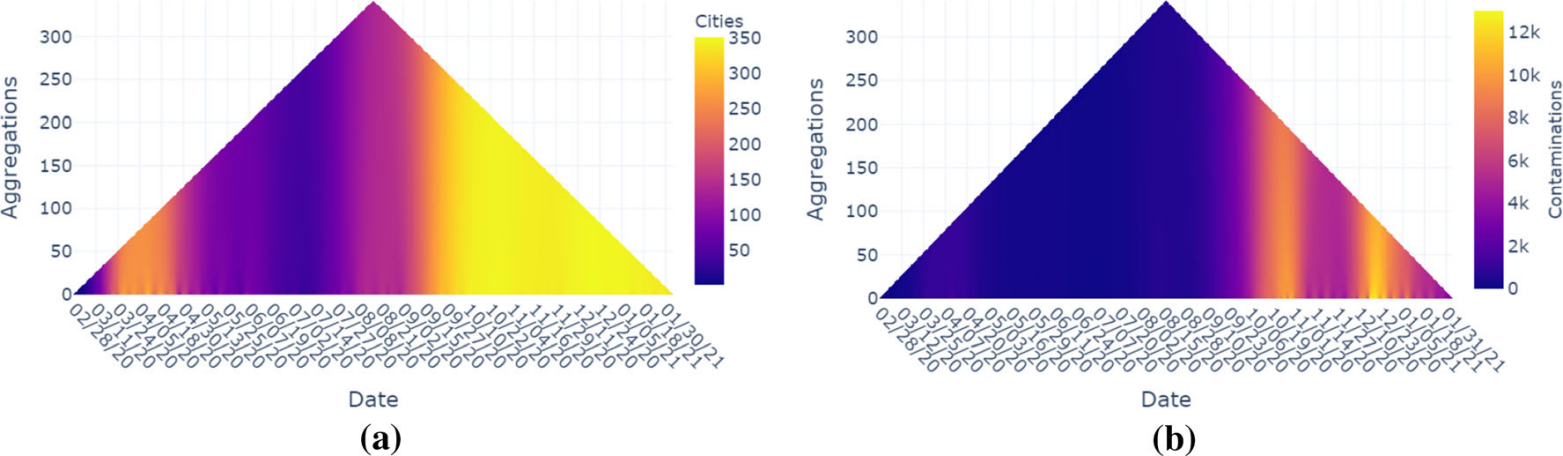
The mean aggregation operator: (**a**) The number of cities that have at least one contamination. (**b**) Total number of contaminations (of all cities)

**Fig. 12:**
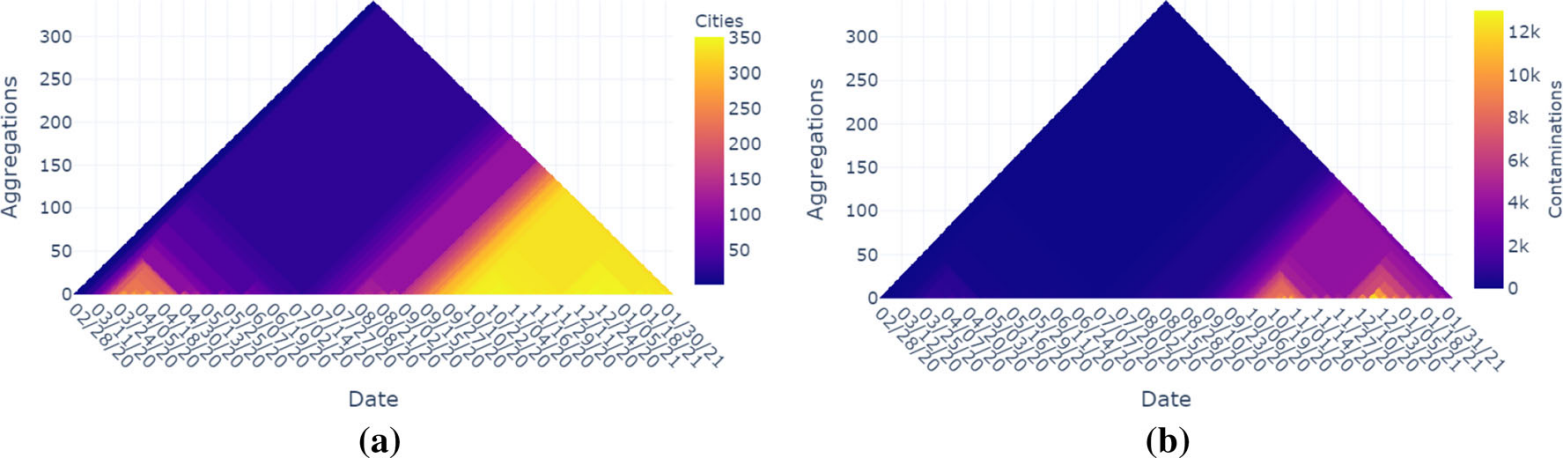
The minimum aggregation operator: (**a**) The number of cities that have at least one contamination. (**b**) Total number of contaminations (of all cities)

What happened during this period? According to the news outlets, the US surpasses 1,000 confirmed Coronavirus cases on March 10, 2020. On March 26, the US leads the world in confirmed cases and many cities issued stay-at-home orders resulting in a dramatic drop in travel. The total flight frequencies in the last week of March 2020 have as a result decreased by approximately 47.4% compared to 2019. To analyze the patterns during, before, and after the Covid-19 pandemic more data are needed.

### Dynamic Covid-19 infection numbers

We applied the heat triangles technique to dynamic Covid-19 data, i.e., each data object at a time point describes the number of cities in the Netherlands with their infection numbers. This results in a time-varying sequence of vectors containing integer numbers for their individual components, i.e., the dynamic data object is a time-dependent vector. Our Covid-19 dataset under investigation is based on the time period starting on February 28, 2020 and ending on January 25, 2021, i.e., the time granularity is on a daily basis.

The first step is to reduce each vector object to a certain reduced number based on a well-defined property. Such a property could be the number of cities that have at least one Covid-19 infection on a specific day, i.e., we count the number of vector components for which the value is larger than 0.

For the first scenario, we use the maximum operator and visually encode in each time subsequence the number of cities in the Netherlands that has at least one infection (see Fig. 10a). Here we see, that in the beginning after a month more than half of the cities have a Covid case. From the beginning of April until the beginning of September, the number of ‘infected cities’ is relatively low, but then starts going up again. From 15th of October on, almost all cities have at least one contamination. In Fig. 10b, we see the maximum number of daily contaminations in any city as a property which gives us a visual peak at the begin of April and one more at the end of October and half of December. Figure 10c shows the total number of contaminations of all cities while the maximum number of contaminations is above 12,000 and is reached at half December. Looking at an aggregation of 50 days (vertical axis), we see that the numbers after a dip in June and July keep on increasing over the whole period.

**Table 1:** Running time in seconds rounded to three decimal places for different numbers of time steps, vertices, and edges

	#Time steps = 1				#Time steps = 10				#Time steps = 100				
	#Vertices				#Vertices				#Vertices				
#Edges	100	1,000	10K	100K	100	1,000	10K	100K	100	1,000	10K	100K	1M
100	4.954				5.273				6.762				
1,000	5.136	5.702			4.900	4.803				6.234			
10,000	5.785	5.792	5.091		5.149	5.174	5.443				6.920		
100,000		7.275	7.466	7.946		7.295	7.207	7.592				9.987	
1,000,000		28.017	26.768	26.081		26.259	26.330	27.981					46.375

Figure 11a shows the number of cities that have at least one contamination with the mean aggregation operator. We can see that there are patterns visible at the lower levels of aggregation. They might be weekly patterns in the testing (e.g., less tests in the weekend?), but that is some kind of hypothesis that has to be checked with another data source and/or another visualization. In Fig. 11b, the total number of contaminations (of all cities) is shown. Here again we see a similar weekly pattern at low levels of aggregation. Again those peaks occur in April (less visible with this colormap), also in October and December.

Figure 12a shows the number of cities that have at least one contamination with the minimum aggregation operator. The diagram somehow gives the results closest to a line graph. The number of ‘infected cities’ increases over the year with peaks in April, October, and December. In Fig. 12b, the total number of contaminations (of all cities) is shown by using the minimum aggregation operator. Here we see that toward the end of the time axis we have a minimum of infection numbers that are still quite high, meaning each city is infected to a certain extent.

## Discussion and limitations

There is still a lot of room left for both improvement and creativity in the further development of the heat triangles, also due to the fact that the visualization is easy to create and easy to understand.

### Loading time

The major drawbacks encountered with the development of the heat triangles so far, however, are concerning the performance, mainly in terms of loading times. Depending on which (other) visualization techniques are desired to be included, the running time might drastically increase or decrease. The scalability of the heat triangle itself, however, depends heavily on which properties are included as well as the type and number of aggregation strategies. The overall loading time was acceptable for relatively small to normal amounts of data, however, for big data such as the US Domestic flights the heat triangle was not able to perform in an acceptable time frame, i.e., the data had to be preprocessed beforehand. To test the scalability of the current implementation, a performance test is carried out on the used method. Table 1 gives an overview about different dataset sizes and the computation time. Here we see that the number of edges has the greatest influence on the overall running time compared to the number of vertices and the number of time steps. In practice, such as the US domestic flight dataset, it is common to have proportionally more vertices and edges when there are more time steps involved. The more time steps, the more vertices there are and consequently, the more edges there might be. Also further aggregation metrics and properties might be considered which also have varying contributions to the running time.

### Interactions and callbacks

As mentioned the heat triangles support several interaction techniques. There is no specific order of which interaction to use first to allow the user to be free in his or her decisions. Every interaction is placed in a position such that it is easy to understand its function. Combined with the simple design of the heat triangle, it is even suitable for non-experts who are not familiar with data analyses and complex visualizations. The designed tool provides the user the ability to have an overview of the data, to zoom in on data of interest at a chosen time, to have details on demand, to view relationships, to go back to the first overview, a previous step or undo the last step and to extract the produced figure as png.

There are, however, still a few points which have to be improved. The pan function for multiple subplots does not simultaneously act for both axes leading to the user having to pan again separately for the other subplot to start comparing. Moreover, the x-axes of the sub heat triangles do not update after a point is chosen in the main heat triangle, leading to the user to have to pan again in the sub triangle when a relatively low point is chosen as the new pinnacle. A log can support the user to replay a sequence of changes. This provides the user with extra insight into his or her data exploration process. Also, the possibility to extract values and time points besides only figures of interest provides the user to store and use these later for further calculations. This way, other statistics, such as the perceptual increase or decrease over a certain period, can be calculated. Many callbacks are generated to not only support the interactions but also to create new figures based on the interactions with others e.g., the sub heat triangle. To do this, the visualization consists of multiple hidden storage places to which the needed data after computation, and transformation is sent in a JSON format and transformed back in a new call back function. This has also been used for other purposes such as avoiding repeated computations, e.g., when another color scheme is chosen by the user, to improve the response time. Many other interactions can be added to the tool, either in one view or to all views.

## Conclusion and future work

In this paper, we introduced the heat triangle, an overview-based interactive visualization for temporally long dynamic data sequences, as a way of abstraction, acting as a powerful first dimension to guide the user to interesting patterns in property-driven data exploration. This is done by computing the property values for each time step and aggregating every two adjacent values by a statistical metric, eventually reaching a triangular shape where the x-axis depicts the time steps, the y-axis shows the level of aggregation, and the values are depicted by the color of the cell. Besides the different views allowing the user to either have an overall overview, to find a relation between different properties, aggregation strategies and periods, the interactions are shown to be powerful, not only enabling the user to extract exact values of information, but also to create additional supporting visualizations. For future work, we plan to evaluate our approach and experiment with further dynamic data scenarios. Moreover, we plan to add more interaction techniques.
